# The Treatment of School Children

**Published:** 1910-01-15

**Authors:** 


### SPECIAL ARTICLE.

### THE TREATMENT OF SCHOOL CHILDREN.

By A SCHOOL MEDICAL OFFICER.

The Education (Administrative Provisions) Act came into force on January 1, 1908. Two years have passed, and, generally speaking, the scheme is in full working order all over the country. Results are coming to hand, various problems have arisen, and future policy and developments are ripe for discussion. The greatest and most difficult of these is undoubtedly the question of treatment. The object of medical inspection is to ascertain whether there were any grounds for believing that there is widespread deterioration in the national physique. What is to be done to prevent or alleviate the defects found is now the question requiring answer.

Several attempts to solve this problem have already been made, notably in London, where it has been decided to use hospitals, for the present at least, in preference to school clinics; in Bradford, where a school clinic has been established; and in Worcestershire, where a voluntary association has been called into being to deal with these cases. Yet another method has been the use of Section XII. of the so-called Children's Charter, which throws on the parent the duty of securing treatment for the child under certain defined circumstances, and points out that if such treatment cannot be provided privately it can be obtained through the Poor Law authority. Some of these plans are tentative, and it is well that they should be. The issues involved do not all lie on the surface, and possibly the framers of the Act foresaw the difficulties ahead when only inspection was enjoined. Certain standards of physique have been established; the common defects are known, and when known they are notified to the parents, coupled with a direction that treatment is desirable. At the same time figures have been obtained which indicate the enormous task treatment by public authority would be. With these facts before us certain questions emerge at once. Should treatment be left entirely to the parents? Should the school authority make itself entirely responsible for it with or without the power of recovery of the cost from the parents? Or can a *via media* be found in which both parents and education authority take a share in the treatment of the children?

Treatment cannot be left entirely to the parents, if it is to be adequately carried out, under present circumstances. Even if Section XII. of the Children's Act is used the duty of parents can be easily avoided. It is open to parents to refuse permission for their children to be inspected, in which case a parent who is aware of or suspects a defect in his child and wishes to evade his responsibility can do so by refusing permission for inspection. Again, in the lowest class parental neglect of children is rampant. In the working classes the expense of treatment is often a fatal obstacle to their doing what they would. Even if medical advice is obtained such conditions as ringworm, scabies, and running ears are rarely adequately attended to at home, and absence on account of these engulfs much school time. In addition, resort to hospitals is very frequent. The percentage of cases of defective vision, for example, treated at hospitals would be an interesting figure if it could be ascertained; and whether this vast duty should be thrown on charitable institutions, and incidentally on a sweated profession, is a question rapidly becoming acute. Yet another objection to parental responsibility for treatment lies in the possibility of difference of opinion between the school doctor and the private medical man. The one may certify a child as fit to return to school after absence from, say, ringworm. The school official, supported by laboratory evidence, may hold very different views, and so, in similar fashion, many sources of difficulty may arise.

Wholesale treatment by the school authority under present conditions is similarly out of the question. Only children in two age groups, "entrants" and "leavers," are inspected. Were treatment provided for them, it would not be unreasonable to suppose that the parents of other children suffering from like defects would come forward and demand treatment for their children. And what of the acute diseases—pneumonia, rheumatic fever, Bright's disease, or meningitis, or even the minor ailments that affect children? Would not parents reasonably expect to secure treatment for these as well as for conditions found in school by the official doctor? Where, then, would the line be drawn? Are those who advocate treatment by the school authorities prepared to treat all ailments of all school children, or do they mean to treat children of certain defined ages and for certain complaints only? Nevertheless, this plan of universal medical supervision of school children in the fullest sense of the term is an ideal which even now is on the way to be realised, but the time is not yet. Possibly reform of the Poor Law may still further pave the way to such an end. Parenthetically it may be pointed out that a recent joint memorandum of the Board of Education and the Local Government Board has placed on the medical officer of health the duty of investigating and taking measures to prevent outbreaks of both notifiable and non-notifiable infectious diseases in schools. For the moment a scheme intermediate between the schemes indicated is more feasible. School managers, the education authority, and parents have all a duty in the scheme we outline.

Where underfed, ill-clad, and ill-used children are found—and naturally they are more numerous in some districts than others—their cases should fall to the care of the local school managers. Their knowledge of local charities and charitable persons, of the particular children and their homes, is peculiar, and should be utilised in any remedial scheme. With the help of the Society for the Prevention of Cruelty to Children, and armed with the Children's Charter, they are in the best position to deal with such cases. The education authority must take a share in the work also, and this can best be done by means of nurses, school clinics, and the special school; but these agencies must work within well-defined limits. Their primary object is to protect the authority from the loss of grant liable to be incurred through the absence of children from school on account of trifling ailments. All children on the school roll irrespective of age would come under their jurisdiction. The nurse's duty would be to deal with cases of uncleanliness, verminous heads, impetigo, and such-like conditions. She would do this by visiting the homes of the children concerned, giving practical demonstrations of how cleanliness is obtained, and exerting a watchful influence on the people to the maintenance of this end. Indeed these duties might well be added to the work of the health visitor now employed so beneficially by many sanitary authorities. In the school clinic her assistance would be required in the treatment of sores, scabies, ringworm, running ears and sore eyes, and generally assisting the staff. The object of the clinic is the treatment of those ailments which, usually neglected by the parents, nevertheless incapacitate a child from school and urgently demand treatment. To this clinic might well be added an eye department. One of the unsatisfactory features of school inspection work is that ophthalmoscopic examination is impossible. In such a department doubtful cases could be diagnosed and spectacles provided at cost price, the price to be paid by the parents. This would in no way injure the private practitioner, as it can hardly be claimed that he gets any considerable share of this work. Certain forms of special school it would also be the duty of the education authority to provide. These include ringworm schools, "open-air" schools, and schools for the mentally deficient and backward. In small authorities the need for these may not be apparent. In large districts the need is all too real. In addition, provision must be made for the blind and deaf.

A ringworm school prevents loss of school attendance on the part of the infected. It also implies treatment. "Open-air" schools are intended for the tuberculous or those of poor physique. Mentally deficient and backward children are to be found in every community. For years they have attended the ordinary school, have been moved from the infant department usually on account of size, and leave at the age of 14, possibly as the lowest placed members of Standard II. The situation is pathetic when the length of time this farce has been played is considered. Attendance is compulsory, therefore these children attend. The duty of attending to them has lain with the education authority, and it remains with it. How much longer will it be ignored?

There are special institutions for the blind and deaf. Into these such children should be obliged to go. But here the limit of the duty of the education authority has been reached. Our idea is that their duty is to supply education for all children, whether they be whole, blind, deaf, or deficient, and that they undertake the treatment of those conditions most frequently causing absence from school and for which children are generally excluded by the school medical officer. As it happens, few cases of the variety under consideration—uncleanliness, itch, ringworm, otorrhœa, etc.—find their way into a medical consulting room. In this way much of what otherwise would mean inevitable loss of attendance would be avoided. All other conditions must be treated by the parents. In selected cases, those in which the agreement of the community would follow action taken, Section XII. of the Children's Act may be put in force. Its regular use would be impolitic. The hundreds of hopelessly carious teeth one sees will probably remain unattended, a festering offence, till pain drives the child to the dentist or chemist. Tonsils and adenoids, such as receive treatment, will continue to get it at hospital where parents are fortunate enough to get hospital tickets. Other conditions will obtain relief, generally speaking, when the child has been put to the test of pain or fever. By these standards do many parents judge of a child's well-being. When treatment is recommended, so often is the reply given, "We shall go to the doctor when it makes him ill." It means many urgent cases will go untreated; but the education authority cannot assume complete responsibility. Reasons for this have been given, but they resolve into one—expense.

